# Support, snacks, and a tailored approach to empower recovery: professionals’ insights on supporting children victimized by online child sexual abuse

**DOI:** 10.3389/fpsyg.2025.1651951

**Published:** 2026-01-09

**Authors:** Anette Birgersson, Linda S. Jonsson

**Affiliations:** Department of Social Work, Marie Cederschiöld University, Stockholm, Sweden

**Keywords:** online child sexual abuse, digital sexual violence, technology-assisted sexual abuse, child sexual exploitation material, trauma, youth, professional support, child advocacy center

## Abstract

**Introduction:**

Even though Online Child Sexual Abuse (OCSA) has become a recognized phenomenon, there are still extensive gaps in knowledge and understanding of the implications of OCSA. A considerable number of children and adolescents (CAA) are affected daily by OCSA, and it has emerged as a growing threat to their physiological and psychological development.

**Method:**

This study aimed to explore the experiences of the professionals working with OCSA in various agencies and roles, inclusive of their perception of how OCSA and their work impacts: Children and Adolescents, Caregivers, and Interagency Cooperation. Qualitative design with an inductive approach were used interviewing 43 Swedish professionals, including: forensic child interviewers, social workers, therapists within child psychiatry, and medical professionals. All had extensive, experience of working with children and adolescent victims of OCSA. The collected material was analyzed using Reflexive Thematic Analysis.

**Results:**

The analysis of the collected material resulted in three main themes and 12 subthemes related to the research questions. The three main themes are: (1) Victim Impact – Feeling Complicit; (2) Caregiver Impact – Sense of Inadequacy; (3) System Impact – System Error. Those interviewed voiced special circumstances highlighting the need for a tailored approach and organizational policies supporting interagency cooperation.

**Discussion:**

Findings bring to light the challenges professionals face managing the lack of policies and national guidelines affecting the support they can provide OSCA victims and caregivers. Participants in the study agree, OCSA victims and their caregivers should be privy to the same protocols applying to children subjected to other forms of crimes. These protocols include incorporating questions addressing potential OCSA, along with routine questions utilized as part of the assessment process in treatment/social service settings.

## 1 Introduction

### 1.1 Background

Online Child Sexual Abuse (OCSA) affects a considerable number of children and adolescents (CAA) daily and has emerged as a growing threat to their development. The effects also negatively impact their physiological, psychological wellbeing as well as impairing their sexual health and relationships (Chauviré-Geib et al., 2024, 2025; Joleby et al., 2024). The adverse consequences of OSCA on CAA, while compared to those of general child sexual abuse (CSA), reveal substantial cross-over in vulnerabilities and impact, although research also show distinct differences in the nature and severity of these experiences (Jonsson et al., 2019). Research suggests that victims of OSCA may experience symptoms that are as severe as, or in some cases more pronounced than those resulting from other types of CSA (Jonsson et al., 2019; Joleby et al., 2020b; Paradiso et al., 2023; Chauviré-Geib et al., 2025).

#### Prevalence

The prevalence rates of OCSA differs depending on what type of methodology is used in the studies. Some recent studies report a prevalence rate between 12.55% to 85%, where CAA under 18 years of age report having experienced some type of OCSA (Landberg et al., 2022; Finkelhor et al., 2023; Childlight – Global Child Safety Institute, 2025; ECPAT Sweden, 2025). The difference between disclosure rates in anonymous surveys via social media online (ECPAT Sweden, 2025; Childlight – Global Child Safety Institute, 2025), and in large-scale national surveys incorporating diverse and representative samples (Landberg et al., 2022; Finkelhor et al., 2023) compared to the number of OSCA cases reported to authorities is high. The discrepancy in prevalence rates suggests that the hidden figures of disclosure and unreported experiences of OCSA are substantial. It is crucial to understand the victims of OCSA and how their vulnerabilities might make them susceptible to being victimized, as well as the effects of disclosing versus nondisclosure.

#### Disclosure

OSCA victims are more often discovered by police while investigating online abuse material than by disclosing the abuse themselves (Knipschild et al., 2025). In a recent study from Joleby et al. (2024), CAA victims of OCSA reported several hindrances for disclosure: conflicting emotions such as feeling unique and alone, fear of not being believed, shame, guilt, and uncertainty about potential repercussions. The online context of the abuse, and the abuse material remaining online, often intensifies feelings of hypervigilance and isolation, which can diminish trust and safety in seeking support from others (Gewirtz-Meydan et al., 2018; Jonsson et al., 2019; Joleby et al., 2024; Knipschild et al., 2025). Society contributes to their reluctance and stigma associated with online abuse, along with concerns about potential misunderstandings by caregivers, significant others, or authorities, appears to significantly influence the decision-making process of children and adolescents regarding disclosure of their experiences (Quayle et al., 2012; Joleby et al., 2024; Knipschild et al., 2025).

#### Professionals’ responses

Research reveals OCSA as having a complex impact. A thorough understanding of the actual prevalence and effects of OCSA seems crucial for identifying various challenges faced by victims and their immediate relationships on several levels; individual, family, professional, and societal (Katz et al., 2021; Lim et al., 2021; Salter and Richardson, 2021; Quayle et al., 2023; Finkelhor et al., 2023). Professionals responding to OCSA represent multiple disciplines, including law enforcement, forensic child interviewers, social protection services, healthcare providers, child advocacy centers, mental health professionals, and others. Each group encounters specific challenges related to their professional role in prevention, identification, investigating, assessing, offering support and intervention for victims (Quayle et al., 2012, 2023, 2024; Katz et al., 2021; Knipschild et al., 2025; Jonsson and Birgersson, 2025). For the multidisciplinary approach to be effective in response to OCSA, collaboration across these professional agencies seems as a central focus, as well as ensuring all involved having specialized knowledge and skills relevant to digital environments and OCSA. In recent years, an increased interest in professional responses has emerged concerning OCSA, both pertaining to treatment, and the experiences of those working with victims and caregivers impacted by OCSA (Quayle et al., 2012, 2023, 2024; Katz et al., 2021; Redmond et al., 2023; Giles et al., 2024; Knipschild et al., 2025; Jonsson and Birgersson, 2025).

Cooperation and coordination of OCSA cases involves not only several agencies, authorities operating under different legal frameworks and confidentiality; it also involves professionals with an array of educational backgrounds and certifications, but also professionals with various personal experiences, values and biases. Coordinating and organizing these differences has shown to present several challenges for professionals, which, if left unaddressed, may adversely affect the victims (Quayle et al., 2012, 2023, 2024; Joleby et al., 2024; Schmidt et al., 2024; Carmo and Manita, 2023).

As the above describes, there is an urgent need for research focusing on the various roles those working with both CAA and OCSA, especially the features of this work that are different and distinct from other forms of harm toward children. This article focuses on the interagency and multidisciplinary approach of the Barnahus system of Sweden. At Barnahus, which functions similar to Child Advocacy Centers, CAA under the age of 18 receive coordinated interventions and support in a child-centered environment if there is a suspicion of physical or sexual harm, after disclosing allegations, and during the legal process. Key professionals including the police, forensic interviewers, social workers, medical staff and clinical therapists each have a role within and conduct their work at Barnahus.

### 1.2 The research questions

This study aimed to explore the experiences of the professionals’ working with OCSA in various agencies and roles. Investigating whether existing interventions are sufficient, or if there exists a need for a more specialized approach to better support young victims and caregivers in their recovery process.

The research aimed to answer the following questions:

- Do professionals working with OCSA experience current interventions and support as adequate to address the unique needs of victims and caregivers?
- Is a more specialized approach needed and if so, what should be addressed?
- What roles do current organizational structures play in addressing OCSA?

## 2 Materials and methods

Utilizing qualitative interviews, the experiences and perspectives of 43 individuals working with OCSA victims was researched through a phenomenological lens. The interview guide included questions focusing on individual experiences and perspectives. The interviews also provided opportunities for reflection regarding their perception of victims’ and caregivers’ needs and vulnerabilities, as well as the support they received after disclosure.

In the text, the following abbreviations are used: Online Child Sexual Abuse (OCSA), Child Sexual Abuse (CSA), Children and adolescents (CAA), Social Worker (SW), Forensic interviewer (FI), Medical professional (MP) and Therapist (TP).

### 2.1 Participants

In the study, 43 professionals were interviewed. These were trained social workers (SW) working within Child Protective Services or at Swedish Barnahus (*n* = 8), Forensic Interviewers (FI) within the police (*n* = 15); Medical Professionals (MP), including medical- and forensic doctors and nurses (*n* = 5); and Therapists (T) working within the Swedish child and adolescent psychiatry (*n* = 15). Inclusion criteria encompassed professionals who had direct experience with victims of OCSA, or who served in supervisory positions within agencies handling such cases.

Participants reported a varied level of experience depending on the number of OCSA cases encountered: forensic interviewers had conducted child interviews with at least 50 children and caregivers; therapists experiencing fewer, but more long-lasting contacts (between 1 and 10 children and caregivers per person); social services saw between 1 and 5 children and caregivers, similar to the medical professionals. Together the participants saw well over 800 OCSA victims. The participants came from cities and regions across Sweden, representing both sparsely populated areas and large metropolitan regions. The participating professionals were mainly women (*n* = 39/43), and male participants were all medical professionals. A majority of the participants professional experience exceeded 5 years within their profession. The number of participants was determined by participants signing up to participate during the project timeframe, 2022–2025.

### 2.2 Design

The study had a qualitative design and phenomenological, inductive approach. Semi-structured interviews were conducted. The interview guide consisted of five main topics, all concerning OCSA victims and their caregivers, to discuss with the participants: Background, Support and Treatment, Parental Support, Collaboration and Ending questions. There were one or two main questions for each topic and further suggested follow-up questions to help the professionals elaborate on their responses. The citations are translated as directly as possible from Swedish to English by the authors.

### 2.3 Procedure

Professionals were initially recruited through different channels using purposeful sampling. Social workers were recruited through the Swedish Barnahus network and groups for social workers on Facebook. Forensic interviewers were recruited through the Police’s National Operational Department (NOA) and Therapists from the Swedish national network for therapists working with trauma-focused therapy Trauma-Focused-Cognitive Behavioral Therapy (TF-CBT), or through groups for social workers and psychologists on Facebook. Medical doctors and nurses were recruited through the Barnahus network and through online advertisements on Facebook. In addition, a snowball sampling strategy was used to cast a wider net. Those who were willing to participate were offered the option of being interviewed online or in person. All participants preferred online interviews, subsequently, the interviews were conducted via Teams. Information about the study along with a consent form was sent out beforehand. At the time of the interview, the participants were informed orally about the study, and that their names, workplace, locations and other identifiable data, such as specific children or legal cases, would be taken and anonymized. They were also informed that they could withdraw from participation at any time. Informed consent was obtained verbally at the time of the interview. The interviews lasted around 1 h each and were recorded via Teams and transcribed verbatim by the researchers. To ensure confidentiality, the transcripts were anonymized and assigned an identifying code, which was also utilized to reference specific excerpts in the Results section. The data was stored securely to maintain participant privacy at the university. Ethical approval for the study was approved by the Swedish Ethical Review Authority, D.nr 2023–08173-01.

### 2.4 Data analysis

The collected data were analyzed through a phenomenological lens using an inductive approach employing Reflexive Thematic Analysis (RTA) by Braun and Clarke (2019, 2022). This dual methodology allows flexibility, and yet gives a systematical framework for organizing, analyzing and identifying patterns in the data both within and across the dataset. The approach opens for the researchers’ subjective interpretation when analyzing the data, allowing a deeper exploration of the professionals lived experiences and perspectives (Braun and Clarke, 2022). RTA includes a six-phase coding framework (Braun and Clarke, 2022), which authors followed. Familiarization of data is the first phase, which includes reading and becoming thoroughly reacquainted with the material, since both authors conducted interviews. In the second phase, the first codes were generated after sorting the main themes, coming up in the interviews, and these then got compared back and forth to the initial data. By inductively deriving the coded data into themes, followed by reviewing the themes several times to determine the significance, with the aim to ensure that the findings mirrored the participants’ perspectives. In the final step, reporting the findings in written text through the authors’ subjective and interpretative lens (Clarke and Braun, 2013; Brown and Clarke, 2019, 2022). The analysis process was interactive between the authors; both being involved in all aspects of the analysis to ensure that the data was reliably interpreted, and that the findings were credible and dependable.

### 2.5 Authors positioning

The main author, AB, is a social worker, MSc, a licensed psychotherapist as well as a PhD student focusing on OCSA. AB has an extensive clinical background, and has specialized in working, training and supervising other professionals concerning children and adolescents with traumatic experiences and/or harmful and or boundary breaking behaviors. AB is also a certified trainer in TF-CBT looks off as well as various other therapy disciplines. ABs experience, interest and clinical engagement in supporting children and caregivers affected by OCSA influences the way she conducts interviews and interpret the data where her clinical eye sometimes shines through.

LSJ is a social worker, associate professor in social work and researcher focusing on child exploitation, with emphasis on OCSA, including pornography use and production among adolescents. She is interested in both clinical and organizational perspectives on sexual violence, particularly in how such issues can be addressed within various organizational settings. She has devoted her research career to sensitive topics and participatory action research making sure research is filling important knowledge gaps and is applicable in clinical practice.

## 3 Results

**Figure 1:**
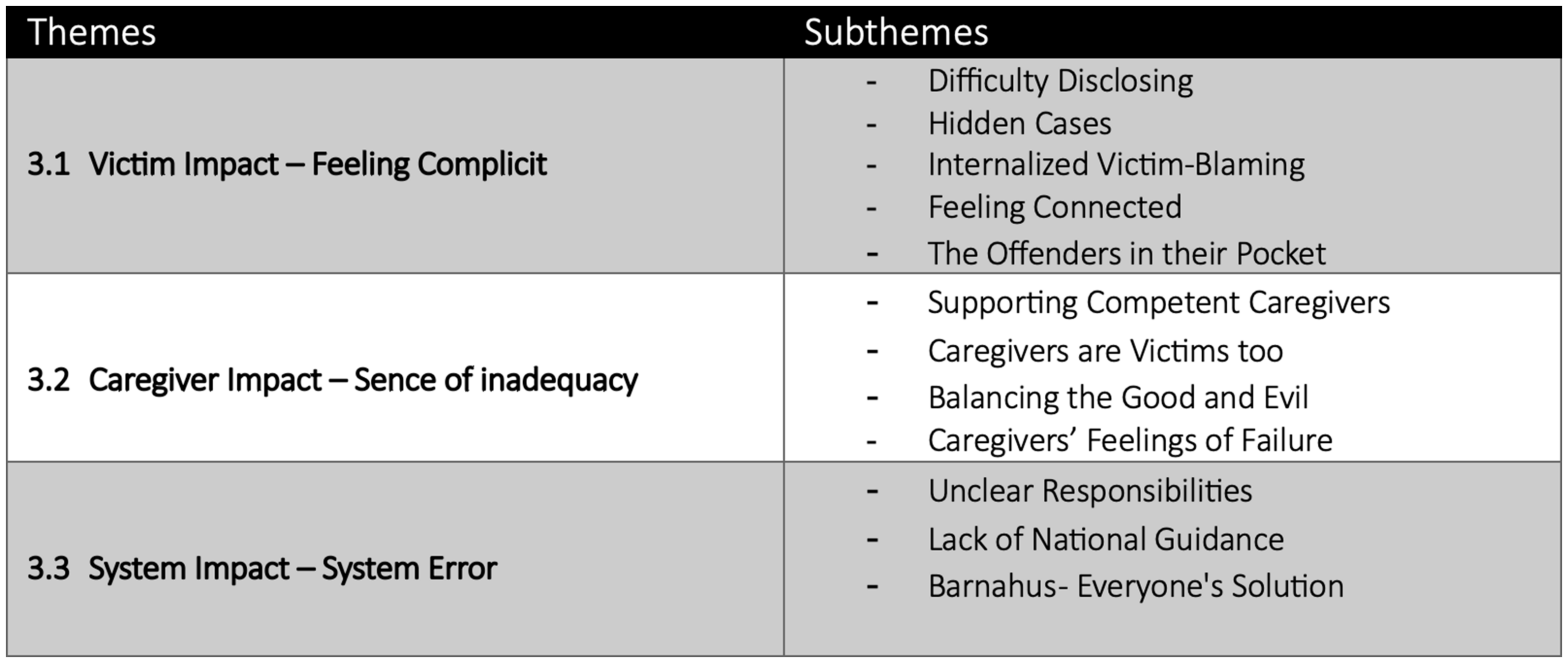
Impact of OCSA - Themes and Subthemes.

In the process of thematizing the data, the professional’s engagement and dedication supporting victims of OCSA and their caregivers shone through. The identified themes reflected not only the diversity of the professionals’ experiences and perspectives but also similarities in the special circumstances and challenges they experience working with OCSA. The data was funneled into three primary themes: (1) Victim Impact – Feeling Complicit; (2) Caregiver Impact – Sense of Inadequacy; (3) System Impact – System Error. Victim impact - Feeling Complicit: this theme and the five subthemes analyze the data concerning the challenges faced by the CAA from the professional’s perspective. They express that the victims’ feeling complicit in the OCSA impacts the entire process from disclosure to recovery. Caregiver Impact – Sense of Inadequacy: this theme shines a light on the complicated position of the caregiver trying to navigate both their emotions and the weight of having to set boundaries with their child to continue as a supportive and effective adult figure. System Impact – System Error: this theme highlights the challenges presented by interagency cooperation, especially with the lack of national guidelines and organization. Each theme represents the experiences and perspectives of those working with OCSA and enhances and broadens the impact of the research questions (Figure 1).

### 3.1 Victim impact – feeling complicit

Most professionals voiced that victims of OCSA, more often than other child sexual abuse victims (CSA), had an overwhelming feeling of complicity in the abuse. They described that regardless how the victim of OCSA was connected to the person abusing them, they often felt complicit in making the abuse possible. This feeling of complicity was described as an overshadowing challenge impacting CAA, caregivers and professionals throughout the process of disclosure to specialized treatment.

But it's a bigger part because there's a lot in it, it's like no one has been in your room. No one held you down and you could have just shut the screen or turned off the phone at any time, and you haven't. Plus, you've been told and warned with the fact that you're not allowed to send pictures, and you have and that's why you're not saying anything to mom. (T, 13)

The more they understand the consequences, the severity, and also their vulnerability, then they can also feel worse, and often they feel worse because they already know it's wrong, they have crossed boundaries, they have talked to mom and dad who told them not to but they still did, so it's usually those feelings that make them feel bad, perhaps more than the abuse itself. But it looks very different depending on the type of abuse. (T, 6)

The participants described a variation in how strong the feelings of complicity were between CAA, often depending on the way they were abused. Their age was also mentioned as a contributing factor. More often, younger victims who were contacted and manipulated to share pictures and/or videos, described feeling less guilt and shame than older CAA. Instead, they might present with other symptoms including being scared, various forms of anxiety and behavioral difficulties. Older victims were reported to often suffer from similar symptoms and feelings of complicity regardless of how the abuse happened. Whether or not the child disclosed the abuse themselves or was discovered as part of a police investigation represents the biggest differences in feelings of complicity.

#### Difficulty disclosing

Professionals often problematized the dangers of being online. For online abuse to occur, the victim must first have access to and engage online, often deciding to respond to messages from other individuals whom they might not know. They found that regardless of whether the offender employs coercion, threats or utilizes validation and flattery, victims of OCSA frequently articulate an awareness knowing they should have refrained from responding. Indicating that those interviewed believed the CAA understood they could have chosen to block the offender or disclosed to an adult since they recognized that the events occurring were inappropriate and wrong. Professionals emphasize that OCSA is never the fault of the CAA. However, they interpret these intentional or unintentional choices to contribute to a sense of complicity within the child, further complicating their emotional response to abuse and their feelings of shame and guilt. They also discuss how complicated it is to balance their words when talking to the victims not to add to the negative feelings they might be experiencing.

Yes, but it's shameful. It's about sex. They know they've done something wrong. That makes it very difficult, I think, for them to talk about it. They know they've done something wrong and it's about sex and these are things that they don't want mom and dad to know too much about. That's why I think it's difficult. (F, 14)

They feel very bad, and I experience that they feel very ashamed, that it is very difficult to tell and who to choose to tell, many tell a friend first and then have a conversation that leads to telling an adult. So, the friends think they should also tell an adult. But above all, they feel very ashamed about having been someone who has been subjected to something like that or has gone along with something like that or with not really seeing that the other person are responsible, that is, the one who has subjected them; they blames themself saying, 'How could I be so damn stupid that I did that? I really knew what was happening. But they fell for these people's flattery and so there is a lot of guilt and shame. (T, 1)

What I experience is that many kids have difficulty talking about what they themselves have done, because the offender makes the children do a lot of things. And in some cases, the children themselves think it is exciting and that they want to do this too, although in retrospect they feel that this was not good or this… well. But that was exciting sometimes. And it gets worse in a way when you yourself feel like you have been involved in this. You feel complicit. (FI, 6)

Professionals underline that the victim’s sense of complicity may be unintentionally reinforced by societal perceptions, environmental influences, and caregivers who do not fully understand the seriousness of OCSA compared to other forms of CSA. This is further exacerbated when caregivers question and do not understand the CAA choice to not reach out to them, despite their assurance of being there to offer support. Often emphasizing through conversations that they have provided information regarding online safety. Professionals find these statements may undermine the magnitude of the situation, leading victims to internalize shared responsibility for the abuse they have experienced.

What is more common with online abuse is that the young person or child themselves feels that they have been active, that they perhaps initially thought they had a flirtation or a romantic relationship, or that they themselves were interested or active. (...) and so dealing with one's own guilt and also, as I said, the surrounding environment's perception of it, that is often a quite large part of it because it often becomes easier for that type of young person to be blamed. (...) "yes, but you were interested, you stayed there, you went there," there is a perceived involvement that causes problems, and I think that needs to be addressed and worked on quite a lot, and as I said, not infrequently you also need to work with the parents' view of it or sometimes even social services. (T, 7)

Therapists often find that treatment for victims of OCSA tends to be prolonged due to the guilt and shame these individuals frequently experience. Recognizing this emotional burden is essential when engaging with and treating OCSA victims of all ages. They also underpin the importance of adapting their treatment when working with OCSA patients.

There's a much greater focus on trying to alleviate the guilt and shame aspect. This is also done in other treatments but usually a shorter intervention. I've probably doubled the time spent talking about whose fault it is and discussing whose fault it is and how it can be, and the division of responsibility in this, and so on. (T, 5)

Professionals often connected the felling of complicity, guilt and shame about what the CAA experienced as a reason for not disclosing and for not reporting to the police. Many think the underreported prevalence of OSCA is extensive.

#### Hidden cases

Disclosure seems greatly affected by the feeling of complicity, and several professionals raise the question of possible hidden figures, that the reported numbers likely not reflect the true scope of a problem. While FI report females representing most cases they see, they acknowledge that recent trends indicate an increase in male victims. This does not correlate with the low numbers of males being reported to the police or receiving support within social services, nor does it correlate with those receiving clinical services as reported by those offering treatment. This leads to questioning how many CAA are affected and never seek treatment. Social services, Barnahus and the Child and Adolescent Psychiatry also report they lack accurate statistics of the number OCSA cases seen, since they are not deemed as a specific type of CSA cases. They believe that this lack of correct data represents a deficit in the system since an accurate reflection of the numbers would raise awareness and show that more resources targeting this group are needed.

In terms of reported numbers, girls are probably in the lead, but I think there's an enormous hidden figure for boys, actually. I really do. (...) I think there's more stigma perhaps if you're a young boy and have sought excitement and then with another of the same sex or so you've explored or contacted or had such contacts with. That it becomes exposing yourself more, that perhaps the parents might have no idea that you like boys or so, and that it becomes a bigger story than the abuse itself. It can also be that boys just shouldn't care, like "screw it, it was nothing" or so. (SW, 6)

Yes, there are probably many reasons, but yes, and then as it's talked about in society, it's often girls and women who are subjected to sexual abuse, and just that can make it difficult for a boy or man to say that I have been, because it's not something that men or boys become to the same extent. But I. I would think that boys are affected much more than we think, at least. (SW, 8)

Professionals are concerned and hope for further research and initiatives to raise the awareness, making it easier for all CAA regardless of gender, gender identity or sexual orientation to disclose and seek help if subjected to OCSA.

#### Internalized victim-blaming

All professionals agree the feeling of complicity, internalized self-blame, causes guilt and shame making disclosure and all interventions from forensic interviewing to treatment more challenging.

It can be very, very difficult to talk about. Even if they talk about the offender in an easy way, they can talk really great and a lot about the offender, everything that they have done, no problem talking. But when it comes to what the child themselves has done, it is. It is very deep inside and there are only short stories or sentences about what they themselves have done and sent material and so on. So, what comes easily in the story, the thing about the offender, you don’t need to ask so many questions because it comes anyway. While you have to work, work, work a lot more with what the child sent themself. (FI, 6)

In cases where the CAA victim has not disclosed the abuse themselves but instead is discovered by the police during an investigation, the reactions is often more challenging, overwhelming and distressing. The routine described by the forensic interviewers are that they call the caregivers after identifying CAA through videos or pictures. In those first calls they cannot disclose any details, they only inform them that the CAA was discovered in an investigation concerning OSCA and they need to schedule an interview. Sometimes the child does not know what it is about when they come for the interview, especially if they maybe have had several connections online.

But for those who have experienced online abuse and decided not to tell, it feels like having their pants pulled down; suddenly everyone has seen this most intimate thing that they have tried to forget, that they have tried to remove from their consciousness. And furthermore, many people find out about it. Social services, the police, perhaps the court, perhaps a therapist. And it becomes really difficult if you actually don't want to. Then it can still turn out well in the end, but it will take a long journey to get there. (FI, 10)

Professionals experience that it often takes longer for many victims to seek help, when they do, they often seek help for other symptoms and disclose the OCSA later. Professionals mentioned that asking about OCSA are not standard during the intake procedure, which also could further delay disclosure.

For children to talk about abuse, which is often a process over time. There are those who tell immediately, but most take some time, think, and come to the conclusion that they want help. And then they are, in a way, ready. Then things can still go wrong when many new people suddenly become aware of what has happened. (FI, 10)

In the cases I have dealt with, online abuse has come to light later, in one case even after we had finished the trauma narrative, and most people have probably come for other symptoms or other traumas, and I have found out about the online stuff later. (T, 2)

We always ask about sexual abuse at the beginning of a consultation, but not specifically online abuse, which I now think we should. (T, 5)

#### Feeling connected

Professionals mentioned a worry of OCSA having a potential influence on the developmental trajectories of OCSA victims. CAA do not distinguish between online and offline interactions anymore, as the internet has become an integrated aspect of their lives and a natural way of connecting and socializing with peers.

That's something I think a lot about. It's about being a young person and exploring identity, especially perhaps young people who feel a bit outside the norm. And those who use the internet as a major source for meeting similar people and so on. That it creates a vulnerability for everyone, but perhaps especially for those individuals. Because the internet is also a very good place for those who want to access children for other purposes. (SW, 8)

Professionals have expressed concerns that OCSA can impact the development of independence, trust and curiosity, which are fundamental for age-appropriate identity formation. This is particularly relevant for CAA who face relationship challenges or wish to explore connections that may fall outside conventional norms. Many professionals across various agencies noted that CAA with neuropsychiatric diagnoses are overrepresented among OCSA victims. Additionally, some have observed that boys and some of the girls they encounter have experienced victimization while exploring same-sex relationships.

It's heartbreaking to me that many of these children I've met and seen are children who are searching for something, searching for affirmation, searching to explore their own sexuality, to understand themselves. To do something like that? Yes, as if they should search a little, maybe not daring to in their own context, or if they don’t really have a any friends, and then they end up in situations like these, and these people exploit that searching and exploring that all young people do, but some might be completely dependent on the internet to express it, and I think it's very sad that they end up being exploited by these people. (SW, 8)

Sometimes, CAA arrive at forensic interviews or treatment sessions without recognizing the abuse that has occurred. They may interpret the experiences as mutual and within the context of a relationship, even when the offender is significantly older or has misrepresented their identity.

Then what is more common in this online abuse is that the young person or child themselves feel that they have been active, that they perhaps from the beginning think that they have had a fling or a love relationship or that they themselves have been interested or have been active themselves. (T7)

Therapists and social workers raise awareness regarding the importance of recognizing that the OCSA could be validating, exciting and potentially sexually arousing for victims. Which can be equal parts confusing if it is involuntary and exhilarating, making the feelings surrounding the abuse more complex, shameful and harder to talk about.

Again, it's about having to work much more with one's thoughts and what one thinks about oneself, and being careful to give, and I think one does this with all sexual assaults, by the way, this thing about giving a little extra psychoeducation about how the body works, meaning how sexuality works. That is to say, even sometimes if one doesn't want something, the body can respond with sexual feelings. For example, a boy can get an erection, a girl can become wet, even if they don't actually want what they are being subjected to, because the body is built in such a way that it reacts to stimuli and is supposed to react to stimuli. (T1)

Several professionals, mostly in the therapist group, emphasized the importance of helping victims understand the function of sexual arousal and that the body can respond even if the situation is forced and involuntary. They also advocated for spreading more knowledge to the public and caregivers regarding this to help raise awareness about how OCSA can affect CAA sexual development if they do not get help understanding how the body can function during sexual stimulation.

#### The offenders in their pocket

The consequences of CAA being constantly online through their devices, are complex and create significant challenges for those around them to fully understand their context, lives, activities and peer connections online. The interviewed professionals emphasized that caregivers lack knowledge of the scope of their children’s online presence and relationships. Many caregivers seem to believe that discussing online safety once or sometimes is sufficient. Professionals describe that they do not think that caregivers ask and talk about what their CAA do online or with whom they are talking too. Additionally, professionals describe the considerable pressure that CAA feels due to constantly being able to be online. A professional described it as *“they always have their offender in their pocket making them accessible through their devices 24/7”* (SW, 8). The offender is present wherever they are. This reality underscores the need for a greater understanding of the impact of technology on the lives of CAA and the ongoing risks they face.

It becomes so different, like, when they have their own phone so early. It's always with them and always on. They always have it, or those who expose them are with them 24/7. That makes it even harder, I think, to just stop having contact. If parents have no control at all, there can be a lot of contact in a short time. (SW, 3)

Professionals describe meeting CAA from the age of 7–8 years having their own device with internet with them at school. The most common platform for OCSA is Snapchat, and they reflect the concern over caregivers letting small children manage their devices by themselves with very little control.

I had a child, small, who had a phone, and I know I sat with this parent and talked about it. Yes, but what control do you have over her phone? No, we can't have that because (...) gets so upset. And there we had a conversation about it, so, but yes, but a tiny, tiny (...) that you wouldn't have let her out at night in (...) or in the city? No, no, no, I'm not crazy either. But that's exactly what you're doing yourself out here in it? Yes, but. (SW, 3)

Adding to the complexity is the continued availability of the abuse material that exists online and the feeling of loss of control, not knowing who will see it, and for how long it will remain online. Therapists mention this as a complicating factor in treatment, since the abuse is perpetuated even after the contact with the offender has ended.

What I think the difference is, is this uncertainty, in most cases, about who the offender is. And there's also been the issue of material being released, or that there's image material that one doesn't have control over, and because one doesn't even know who the perpetrator is in several cases, one can't really, so it's much harder to feel protected. That is, I think it becomes a greater challenge to work with external stability, external stabilization, given that it is a fact that one cannot protect oneself in that way. The material remains. (T, 8)

Several therapists mention the continued availability of the abuse material online as an on-going trauma for the CAA. They come to treatment and work with traumatic experiences that in some ways still are going on, which complicates the treatment and treatment success.

### 3.2 Caregiver impact – sense of inadequacy

Professionals observe that caregivers often experience distress and shock, and their reactions can be understood as crisis responses. However, most caregivers can cope effectively and do their best to support their child who has experienced abuse. Many caregivers struggle with the realization that their child has been abused online. In their effort to understand the situation, they may sometimes appear to blame the victim by asking questions such as: ‘How could this happen? We talked about this; why did not you tell me?” This reaction is often more pronounced among caregivers who learn about the abuse when forensic interviewers inform them that abusive material has been discovered during an investigation than when the child disclosed the abuse themselves.

Then it becomes a slightly different approach, where we might call the parents and say "hello, we suspect your child has been subjected to a crime." And that can also lead to very mixed feelings, where some parents just say "no, absolutely not," but some become very upset and think it's terrible, of course. And then you have to talk to the parents, inform them, maybe hear what they know or can only suspect, and then we talk to the child. (FI, 12)

Then I also think that the parents' shock over what has happened, I almost perceive it as even greater, and I think that has to do with the perception that children who have made contact with someone online have somehow taken their own initiative, or exposed themselves to things, and that can be very tough. You have a young teenager whom you haven't really seen as a sexual being. They are an innocent child, and then you discover that they are engaging in quite advanced sexual activities online. That can be very difficult for parents, and you really need to talk about it. (T, 10)

Professionals express similar experiences of caregiver’s reactions although a slightly diverse view of how extensive and by whom the caregivers should receive support. Unclear roles and responsibilities between agencies and authorities becomes visible.

I always do interviews at Barnahus, you know, because then I know that the caregivers will have someone other than me to talk to. I don’t have the training or time like the Barnahus staff has. I wish social services always was present, but they rarely show up even if we always report to them. (FI, 8)

#### Supporting competent caregivers

Professionals from all agencies report that competent caregivers and their children typically receive less support, even though almost all cases are reported to Social Services. Even when these cases are brought to the attention of the appropriate agencies, they often only reach the pre-assessment phase at Social Services due to the perception that the caregivers are effectively supporting their children. Several participants from various groups suggest that this could be a significant factor contributing to the trend of children seeking psychological assessments for issues that may stem from unmet needs.

So no, I completely, well, often hear, no, but we don't open an investigation here because the parents seem so competent. They've also made a police report. Yes, but there's nothing that says they can manage to be there and support and, well. (SW, 3)

Social workers, as well as other professionals, noted the lack of presence of Social Services during forensic interviews and subsequent support.

Again, it's very young children who have been exposed, and then puberty becomes a time when these issues resurface. And this memory, you see it with new eyes, with your maturity. And there I can feel that, like, has this family had the support they need so that they can care for this and manage this, like, so I think that on the one hand we should be in more cases and on the other hand we are and should be the ultimate protection, like. If the police and other ones meet these families think that they are very adequate parents. They have supported them. They have done, like. We have no worries here. No, then maybe it shouldn't come to us, so I'm a bit torn about it. I think on the one hand that it is we who should assess it, but I think on the other hand. We can't do everything in either, our cooperation partners are also able to decide in their own agency. (SW, 8)

The unclarity in the role of Social Services concerning OCSA cases is seen as a central issue by all professionals no matter profession.

#### Caregivers are victims too

Professionals generally describe two main types of scenarios regarding how cases are initiated: caregivers, another relative or adult close to the CAA, school staff, or Social Services report abuse to the police, or the child is identified in an ongoing investigation of OCSA by the police. Caregivers are generally described as being in a state of shock and crisis when the forensic investigators make contact. However, cases in which the child has been located by the police tend to be particularly distressing for caregivers.

Because those who report themselves, they are... they want to report. They feel that this was wrong, and I want the police to be aware of this and investigate further. The others are not infrequently children who have not dared or wanted to report. And then it becomes a bit of a surprise for them when you call and have to deliver that difficult news that we suspect. Yes, or to the guardians, that we suspect your child has been subjected to a crime and so on. (FI, 13)

Caregivers’ initial responses differ, and professionals describe most caregivers being help-seeking and in shock when they have the first contact, usually over a phone call. It is often evident that the caregivers grapple with understanding how this could have happened to their child. Professionals emphasize that caregivers might also experience feelings of guilt and shame when finding out what happened.

It's quite difficult, and not infrequently, I think that this type of abuse is perceived not only by the victim themselves but also by their surroundings as "yes, but you yourself were the driving force, you yourself did things," which also means that it's often a rather long process to get the parents to understand the child's situation, not to blame, not to question, etc., because that's what the surroundings do, and as a consequence, the child themselves. So it's often a long process to figure out whose fault it is, where the responsibility lies, how to understand what the child has done or not, so it can be quite a painful process, and precisely this idea that we think children often need, even if they are an older teenager, they somehow need to have their close relatives and family with them, and that takes some time. (T, 7)

Some professionals find that the caregivers struggle with learning that this has happened in their own house, maybe even when they were at home and still the CAA did not reach out or asked for help.

Getting to know, you know as a parent, that your child has been sexually abused online maybe in the next room at home, when I was watching tv. It must be so awful. (FI, 5)

Caregivers reported feeling helpless, finding it very difficult to know how to help their CAA, and what they could have done to prevent this from happening again.

#### Balancing the good and evil

Caregiver’s heterogeneities are described through interviews. Caregivers come from diverse nationalities and various socio-economic groups are represented. Although one common factor is mentioned by the professionals: the lack of adult support and communication with caregivers for OCSA victims. The relationships between caregivers and children are not described as poor, although almost all victims are described as very lonely and not very communicative with their caregivers.

Professionals, raising concern for the potential perception of caregiver-blaming, emphasize the difficulty for caregivers to offer support and coaching to their children when dealing with online connections and relationships.

What the parent does afterwards and how it turns out is everything, and we've encountered everything. We've encountered parents who have forbidden all forms of internet and phones, and we've had parents who have shamed, like if the daughter herself or the son himself sought out this man or person or whatever, and there's a lot to work to do there, and it would need to be coordinated much more. (SW, 8)

Professionals also differentiate between young children and adolescents. Adolescents tend to pull back from their caregivers, which is typical for their developmental stage. As an adolescent, it is normal to begin to separate from their caregivers and, in turn, caregivers then tend to know less about their child. Although, when caregivers find out that their children have had contacts online which resulted in abuse, the reactions sometimes are described as punitive, even if professionals say they can understand that the reaction comes from fear and worry, they stress that increasing control and taking away devices from adolescents can bring more harm than protection and not achieve the intended results of reducing harm.

(...) I think these were parents who had a rather great need for control. They wanted full control and couldn't understand that children needed to test and try things and not always do what our parents say, (...) And their solution was to take everything away. She wasn't even allowed to have her phone. She wasn't allowed to have anything, they just took everything from her, and she was, I mean. She wasn't 7, she was like 13, 14, and, well, her life, she's on her way out, we have to. (...) I could hear how they made it impossible and tied knots in their relationship, because you can't lock her in either. She's on her way, but they were very, very worried, so I can feel a great deal of anxiety about the unknown, about daring to let go about daring to trust again, because she has lied once or twice. Or does the focus become that she has done wrong? Not that she is vulnerable, that's exactly what happened, and she became very locked in. This girl, and I mean, in a way, almost became so that I understand why she lied. What else was she supposed to do? She also needs to start her life, but it was almost impossible to get the parents to see that, yes, but you have to dare. (SW, 3)

Participants in the study stress the importance of professionals and caregivers remaining calm and sensitive to the CAA developmental needs when interacting with CAA and those that care for them.

The internet is an important part of young people's social life. So, I think a lot about the balancing act when I consider that, like, not to negate the good in order to protect from the bad, that it's that balancing act both parents and we need to be in. (SW, 8)

Professionals describe the difficulty in supporting caregivers while setting boundaries without adding to shame and guilt is similar to the balancing act that caregivers face when supporting their child.

#### Caregivers’ feelings of failure

Most professionals express concern regarding the difficulty reaching and supporting caregivers, especially immediately after a disclosure of OCSA. They suggest that providing initial support to caregivers can reduce feelings of shame and guilt associated with being a caregiver to a CAA victimized by OCSA. This support may enable caregivers to assist and communicate with their children more effectively.

Parents have a bit more difficulty with this, in that they feel they have talked about internet safety with their children, and yet this has happened, and it creates some kind of conflict for the parents, not that they mean to blame the child, but they do when they say so, even if they don't mean it. I think many parents feel ashamed and like failures, and then when they get upset, what they say sounds wrong, becomes accusatory, so to speak. (SW, 1)

“We often need to help support caregivers and help them understand what happened. Sometimes it feels like they are embarrassed that it has happened. Like they are bad caregivers. Thats also why I think it’s good to have the interviews at the Barnahus, because they are good at talking to the caregivers so I can concentrate on the child interview. (FI, 11)

The professionals have consensus regarding the caregiver’s need for support through the process from disclosure of OCSA to potential treatment, although there are some questions concerning who and what stakeholder should offer that support.

### 3.3 System impact – system error

Most professionals acknowledge that OCSA should no longer be viewed as a new phenomenon. However, they also express concerns that their agencies have not adequately organized themselves to effectively support OCSA victims and their caregivers.

I think it's good that this is being brought to the table, because as I said, I've thought about it before too.(...) That many of us are fumbling like that, and I think it's good that it, well, as you say, that it's being brought to the table and that someone is taking a grip on it, because this is nothing new. We've known this for a long time. (SW, 2)

They stress the importance of both the CAA and their caregiver being offered the support they need as soon after the disclosure as possible to potentially prevent suffering and lingering trauma symptoms.

#### Unclear responsibilities

Professionals within Barnahus have highlighted the challenges posed by their lack of official responsibility for OCSA cases, as this type of abuse is not explicitly recognized as part of their target group, even though OCSA is a form of sexual violence making it inclusive of Barnahus services. This absence of recognition and clarification results in the inability to maintain comprehensive statistics on OCSA cases, thereby complicating efforts to advocate for the inclusion of OCSA within their target group.

Since they are not part of our target group, we can’t see how many of our sexual abuse cases are online. I guess we could have our own statistics, but I haven't thought about it before, but we should then we can maybe get them to belong here. (SW, 6)

Yes, and I think this is something that will only increase. We see, I mean, if you look at general statistics, you see that sexual contacts for young people in reality are decreasing, while sexual contacts online are becoming more common. So, if we are now only talking about the usual, like natural normal sexual development. It's increasing online, which will also mean that there will be more. So, no matter what we do, it will. It will be a growing phenomenon and. And we have to find ways to deal with it, then the question is how quickly one can adapt to it. (SW, 3)

Furthermore, the specialized law enforcement units managing OCSA cases are small units often operating outside the existing Barnahus collaborative framework. This has been identified as a significant barrier which undermines effective responses to OCSA.

We very rarely receive those cases, I believe, or we don't have an established collaboration with the group that investigates these cases. Not in the same way as with the group that works with domestic violence. (SW, 1)

We hardly get any referrals, not for health examinations or forensic examinations. This is problematic since we can’t operate on our own. We need a decision from the prosecutor to do the forensic interview, and the parents need to consent for a health examination. So even if the child really needs medical support, the lack of us working together hinder the child from receiving support. (MP, 4)

There is significant variability across cities and regions in the country regarding the organization and collaboration surrounding OCSA cases. In some regions, professionals have integrated OCSA within the Barnahus. This seems to be the result of initiatives and connections between individual professionals without official decisions or contracts between agencies and authorities.

Even if they aren’t in this house, they come here for the interviews and we have multi-agency meetings just like in other cases concerning kids, it’s just how we do things and we always have social services in the meetings, but they don’t always come to the interviews. (SW, 2)

In contrast, many others indicate that collaboration in OCSA cases often is insufficient and more challenging to implement. Specifically, the lack of interagency meetings has increased.

Over the past 2-3 years, the number of children who have been interviewed with this problem has increased more and more with us. But we have never had multi-agency meetings, and our agreement that was written when we created the children's house here with us was written (...) or just before that, and it has never been rewritten, so it's like that. It's in the plan that we should rewrite it, and then one of the question marks is, shouldn't we include grooming or online abuse crimes? (SW 4)

The view of who does what and how well the interagency cooperation functions describe being fully dependent on individual professionals and their connections with professionals within the other concerned agencies and authorities.

#### Lack of national guidance

The professionals have clearly articulated the variations in the handling of OCSA cases across different regions and municipalities. A shared concern among professionals in the field is the absence of standardized national guidelines related to the organization and accountability in managing OCSA cases.

We work very differently, all the Barnahus. I have understood, we work in very different ways. We don't have one, what's it called, an authority above us that holds our Barnahus together. (SW, 1)

OK, then we have to increase resources. Then we have to fight for it. (…) We have to change focus, (…) "what is needed?" Then that's what we have to work with. I think we should change focus and we have to, we have to, like, wake up and smell the coffee? Yes, but we are here, this is not a new phenomenon, the children are there and we must help them. (SW, 3)

In regions and municipalities where Barnahus and the police have established collaboration, the specialized units within the police investigating OCSA tend to prefer conducting interviews with CAA at the Barnahus facility. This practice often results in families receiving support services from Barnahus, even though they are not officially recognized as part of the target group.

We have organized it so that the OCSA group here in (...) then receives the cases that are online, so we consult about these children, and a plan is made (...) in connection with the police interrogation, we offer support from Barnahus. For many of these cases or these children, no investigation is opened by social services. Sometimes you can do that depending on the circumstances if the child seems to have great needs. Otherwise, it's actually up to us. They are then referred for follow-up and support after the police investigation has started. (...) We always make sure to contact social services before these consultations and ask, do you want to participate to make an assessment? And we think it's actually good if they do, at least so that they can make a preliminary assessment based on a little more than just the paper from the police. We have varying success, but there are some municipalities that always come. At least they listen in and perhaps make more well-founded decisions, I think. Then there are some municipalities that think that we at Barnahus can just as well make that assessment in that case. (SW, 5)

Concerns regarding the lack of involvement from Social Services are raised by all professionals, including some working within the social services. Inconsistent involvement is raised also in regions and municipalities where cooperation seemingly is established.

But we actually believe that it might have been sensible for social services to be involved from the beginning and meet the family, and that the investigation should have been conducted to a greater extent or more times. Parents can be very competent in the moment but still need help and support afterwards. (SW, 5)

So, we always make sure to contact social services before these consultations, asking, do you want to participate to make an assessment? And we actually think it's good if they do, at least so they can make a preliminary assessment based on a bit more than just the paper from the police, so to speak, and we have varying success, but there are some municipalities that always, always come. At least they listen in and perhaps make more well-founded decisions, I think. Then there are some municipalities that think that we here at Barnahus can just as well make that assessment. (SW, 2)

Some professionals within Social Services report that the cooperation and organizational structure are operating as intended. They note that the existing collaboration meets the needs of support services to families stressing that social services only should be active when caregivers are not able to support their child, or when there is concern for the child’s safety and/or development.

Then I also think that when we receive this type of report, it may rather be that a police report has been filed and then the police send us a SOL 14. Where we can then confirm that yes, there is a plan with the police, meaning that the parent in question has themselves tried to get to the bottom of it and tried to act, and those cases insofar as the child does not show obvious symptoms and we become worried for that reason. But if it is a guardian who guides the child and does what they should and has the adequate contacts required, then it is usually nothing we initiate.

(SW, 7)

While some professionals are more divided regarding how it should work.

But my only thought is, so many of these cases that were at Barnahus, and so few such cases, I feel, go through here? Because it's, it's not the usual cases that we sit here and investigate children who have been subjected to sexual abuse on the internet. So there I feel like, no, no, I would think that not everyone reports concerns to us, even if they perhaps should. Or they do it if one makes preliminary assessments where one sees that the parents have control of the situation and one does not initiate an investigation, that could also be it, but my, my experience is that when those interrogations were at the Barnahus I was at. I can't see that there was always a social worker present in this. Actually. (SW, 8)

According to the professionals in this study, the lack of clarity and organization increase the risk that families who require increased support will fall through the cracks of the system and not receive the help they need.

#### Barnahus – everyone’s solution

Professionals across various agencies appear to prefer involving Barnahus in these cases. Notably, several Barnahus, along with police, social services, and child psychiatry, have incorporated OCSA in their practices, even though it is not reflected in their official policy documents.

I am mainly at Barnahus in (...) because it is the only one, we have (...). It is usually a calm environment; we are not a big city so there isn't much going on there. (FI, 11)

We prepare fika, juice, and buns, everything as we always do before an interview. And when they arrive, we always go in. We don't care if we aren’t supposed to have a consultation; if we have the opportunity, we always go in and talk to them beforehand and ask if there's anyone we can go in and talk to. Is there anyone who might need some support? So, if we have the opportunity, we always do that, even if we haven't had a consultation or if the case is not ours. (SW, 3)

We always do the interviews at Barnahus, even if we it’s not officially said so, so it just is better. Their rooms are better, and it feels better for the families, and they have better coffee and cookies there which the kids like. (FI, 15)

Professionals emphasize that collaboration between agencies significantly benefits families. They describe how cooperation enables professionals to support one another in focusing on their specific tasks, thereby enhancing the approach toward CAA and caregivers. Additionally, this collaboration improves and streamlines work procedures, leading to greater overall efficiency.

Now we have had Barnahus here for many years. We keep them there, in a slightly more relaxed environment or so, and the staff are good and help with fika and can talk to the parents while we interview the child so I can focus on the interview. (FI, 10)

Conducting interviews at Barnahus is described as a more comfortable experience for everyone involved, especially for the CAA. All professionals highlight that the environment helps children relax and feel safer. Simple gestures, such as offering snacks and beverages, contribute to making the experience feel less frightening for the children.

So, then they come to us, we meet up at Barnahus, show them the premises. They inform a bit and offer snacks, and it is a calmer and more pleasant environment there. (FI, 12)

## 4 Discussion

The themes represent commonalities over the professional spectrum indicating that their views, insights and experiences are very similar, even though they see OCSA victims and caregivers in their respective role and context. The findings align with other research, although the professionals’ experiences of distinct challenges and differences faced by victims of OSCA revel a broader picture of the complexities impacting their work (Quayle et al., 2012, 2023, 2024; Katz et al., 2021; Redmond et al., 2023; Giles et al., 2024; Knipschild et al., 2025; Jonsson and Birgersson, 2025).

In the result section, themes were organized hierarchically according to the impact of OCSA, as perceived by those interviewed. Those within the four different professions stress mutual worry and difficulties in relation to CAA and caregivers, and somewhat different concerns regarding theme three (System Impact – System Error).

### Children and adolescents

Professionals express a significant concern for OCSA victims and their developmental, physiological, psychological and relational wellbeing while describing recognizing the impact from OCSA found in other studies (Jonsson et al., 2019; Chauviré-Geib, et al., 2024, 2025; Joleby et al., 2024). CAA impacted by OCSA seem to display significant difficulties not only regarding online victimization but also concerning social functioning and other mental health issues. Several professionals mention CAA with neurodevelopmental differences, CAA exploring sexual identity and CAA that have been exposed to traumatic experiences earlier in their life and or over a longer period over time (Jonsson et al., 2019; Finkelhor et al., 2023; McInroy et al., 2019).

Apprehension is voiced by professionals regarding the developmental identity process of CAA and the potential disruptive impact of OCSA. During puberty, it is increasingly common for children and adolescents to explore social and romantic relationships online (Dienlin and Johannes, 2020; Hoehe and Thibaut, 2020; Saint-Eloi Cadely, et al., 2020). Being able to search and find information tailored to their own interests and finding connections with likeminded peers, perspectives and sexual orientations/expressions is both age typical and positive behavior for many (Valkenburg and Peter, 2011; Lykens et al., 2019; McInroy et al., 2019; UNICEF Innocenti, 2025). However, being targeted and exploited during this process, within these online spaces poses significant challenges, exacerbating their barriers to full disclosure during forensic interviews as well as hindering their ability to seek and accept support and treatment (Manrai et al., 2021; Joleby et al., 2024). Several professionals recognized the dilemma managing CAA’ desire of being online and the risk and often worry about re-victimized. Professionals emphasized being online as an age typical behavior, however when CAA then are victimized through OCSA, difficulties mitigating the balance when talking to the CAA and caregiver not focusing solely on the risks online became apparent. Professionals were extremely aware of and afraid of coming off as victim-blaming, adding on to the guilt and shame the CAA already often feel, knowing that this might hinder further disclosure and recovery (Joleby et al., 2020a, 2020b, 2024; Katz et al., 2021; Priebe and Svedin, 2008, 2012).

Feeling complicit in the OSCA is often experienced by CAA as a significant concern; professionals interpret this phenomenon as contributing feelings of shame and guilt. Such emotional responses can discourage CAA’ willingness to disclose their experiences and seek support, ultimately prolonging their suffering and delaying potential recovery through the acceptance of therapeutic interventions and appropriate treatment modalities (Gewirtz-Meydan et al., 2018; Jonsson et al., 2019; Joleby et al., 2024; Knipschild et al., 2025). The CAA feelings of complicity are present in almost every victim the professionals encounter, although the CAA that have chosen not to disclose themselves appear to struggle even more with accepting other perspectives and assigning blame outside themselves (Hamilton-Giachritsis et al., 2017; Joleby et al., 2020b). Therapists raise this as a major theme in treatment that pinpoints the need for individualized treatment tailored for OCSA victimization (Jonsson et al., 2019; Butterby and Hackett, 2021). Therapists describe the need for adapting and enhancing their approach, assessment and interventions (Dimitropoulos et al., 2022; Quayle et al., 2023; Doyle et al., 2024). Adding psychoeducation to better understand the body’s sexual functioning is necessary for understanding and recovery as well as adding sexual health as part of the treatment (Cohen et al., 2006; Knipschild and Bicanic, 2018).

Caregivers’ responses are often challenging for professionals, necessitating a careful approach to avoid reactions that could worsen feelings of self-blame for both victims and caregivers. Caregivers’ reactions during and after disclosure of OCSA differ widely (Whittle H. C. et al., 2013; Katz et al., 2021; Hall et al., 2023).

Professionals understand that these dynamics can make it more difficult for child victims to disclose their experiences and disrupt their recovery especially if they react by either not acknowledging that it might be harmful, or have happened at all, or if they point out to the CAA that they have talked about this over the years and that they should have blocked the person and come and told them what was happening. Professional insights resonate with research that has found victims might hesitate to disclose being afraid of different types of repercussions such as; caregivers not believing them or blaming them for what happened or not being allowed to be on online (Priebe and Svedin, 2008; Priebe et al., 2013; Whittle, H. et al., 2013; Katz et al., 2021; Joleby et al., 2024) Moreover, professionals recognize that negative interactions can adversely affect the relationship between caregivers and children, which is crucial for effective support and healing. They also emphasize the importance of addressing caregivers’ crisis reactions and potential trauma responses, as the caregivers’ reactions often indicate that they exhibit significant symptoms. These insights are congruent with findings in a finish report where they found caregivers reporting almost as high trauma symptoms as the impacted CAA (Leivo et al., 2024).

### Caregivers

The support needs of caregivers reveal differences in perspectives among various professions and agencies, leading to discussions about how they can better assist caregivers both as a person in potential crisis and in their parental role. Professionals stress the importance of recognizing the specific roles that different organizations play in offering this support and how they can cooperate between agencies to ensure the needs of both caregivers and CAA are met (Quayle et al., 2023; Phippen and Bond, 2023; Jonsson and Birgersson, 2025).

### Professionals

A shared concern amongst the professionals is the absence of standardized national guidelines pertaining to the organization and accountability in managing OCSA cases. This lack of coherence in policy may obstruct effective responses and interventions. Many professionals emphasize the necessity for a unified approach to ensure the safety and wellbeing of children, adolescents and caregivers (Slane et al., 2018; Lindenbach et al., 2022; Quayle et al., 2023; Knipschild et al., 2025; Jonsson and Birgersson, 2025). Additionally, professionals stress the rationale and the importance of several factors, such as conducting the forensic interviews at Barnahus, and the importance of meeting the CAA and caregivers in a homelike setting complete with snacks and incorporating a trauma-sensitive approach. This approach is utilized regardless of the formal inclusion in the Barnahus target group, leading to concerns that statistical data is not accurate in reflecting the number of cases encountered, limiting advocacy for structural changes (Phippen and Bond, 2023; Joleby et al., 2024).

The findings presented in this article highlight a consensus among these professionals on the necessity to adapt their approach and structure to OCSA cases. Also highlighted are the challenges professionals face in managing the lack of policies and national guidelines affecting the support they can provide OSCA victims and caregivers. Professionals agree that OCSA victims and their caregivers should be a part of Barnahus target group and follow the same protocol as other children subjected to crime. This should include asking for information related to OCSA along with other routine questions CAA and caregivers receive upon entrance into treatment (Schmidt et al., 2024; Knipschild et al., 2025). Lastly, the importance of meeting the children, adolescents and their caregivers with a trauma-sensitive approach in a homelike setting, while addressing their needs and vulnerabilities is key. CAA and caregivers need a safe, calm and inviting setting including getting their basic needs met through having snacks and drinks available before, during and after meetings.

The study participants indicated a specific lack of understanding of impact for those that question their identity, and those that have neurodevelopmental differences. Also, they indicated that since female victims are overrepresented in data, future studies specifically on male victims is also needed. Studies aimed at resolving organizational gaps and finding solutions resolving the challenges identified by professionals through this study would also positively impact the treatment for OCSA.

## Limitations

Even if the study highlights important findings on professionals’ experiences of working with victims of online sexual abuse, the study has limitations that need to be addressed. First, the study lacks insight from children and adolescents themselves. Neither are caregivers’ voices represented that might have given important perspectives regarding support and interventions. The perspectives and experiences of victimized children and adolescents and their caregivers are still understudied and something that requires attention in future studies. The study involved various professional groups, though not the same number in each group. For example, medical professionals’ voices were not featured as prominently as therapists or forensic interviewers.

## Data Availability

The datasets generated and analyzed during the current study are not publicly available due to the presence of sensitive information that could compromise participant confidentiality. Data access may be granted upon reasonable request and with appropriate safeguards; inquiries should be directed to the corresponding author.
